# Is Transcranial Direct Current Stimulation Effective for Cognitive Dysfunction in Substance Use Disorders? A Systematic Review

**DOI:** 10.3390/brainsci14080754

**Published:** 2024-07-27

**Authors:** Xinbi Zhang, Mingming Huang, Ying Yu, Xiaoke Zhong, Shengyu Dai, Yuanfu Dai, Changhao Jiang

**Affiliations:** 1 The Center of Neuroscience and Sports, Capital University of Physical Education and Sports, Beijing 100191, China; xinbi.zhang@hotmail.com (X.Z.);; 2 School of Kinesiology and Health, Capital University of Physical Education and Sports, Beijing 100191, China; 3 Key Laboratory of Sport Training of General Administration of Sport of China, Beijing Sport University, Beijing 100084, China; 4 Sports, Exercise and Brain Sciences Laboratory, Beijing Sport University, Beijing 100084, China; 5 School of Physical Education and Sport Science, Fujian Normal University, No. 18, Wulongjiang Middle Avenue, Shangjie Town, Minhou County, Fuzhou 350108, China

**Keywords:** substance use disorders, cognitive dysfunction, transcranial direct current stimulation, tDCS, cognitive function, non-invasive brain stimulation

## Abstract

Patients with substance use disorders (SUDs) often suffer from cognitive dysfunction (CD), affecting their quality of life and daily functioning. Current treatments, including pharmacotherapy and psychotherapy, have limited efficacy and notable side effects. Transcranial direct current stimulation (tDCS), a non-invasive technique that modulates cortical activity, shows promise in improving cognitive function with minimal side effects and low cost, and could potentially serve as a valuable adjunct to existing therapies. This systematic review aims to evaluate the literature on the effectiveness of tDCS for CD in SUD patients to inform clinical practice and future research. Following PRISMA guidelines, the review includes studies that used tDCS for SUD-related CD. The criteria for inclusion encompassed participants aged 18 and older with a diagnosis of SUD, the use of tDCS (either conventional or high-definition), control groups receiving sham stimulation or no intervention, and cognitive outcome measures for substance-related cognitive function using validated tools. Databases searched were Ovid MEDLINE, PubMed, Web of Science, Embase, Scopus, and PsycINFO, with specific keywords. Twenty-two studies met the criteria, suggesting tDCS can improve cognitive functions in SUD patients, though results varied. Effectiveness may depend on the brain area targeted, stimulation parameters, task requirements, and individual differences. tDCS shows potential in treating SUD-related CD, but further research is needed to optimize stimulation protocols and address study variability. Future studies should use functional magnetic resonance imaging to explore the brain mechanisms by which tDCS improves cognitive function in SUDs and focus on larger, long-term trials to confirm efficacy and refine tDCS treatment parameters.

## 1. Introduction

Cognitive dysfunction (CD), characterized by decline in memory, attention, executive function, and language abilities, significantly impacts patients’ quality of life and daily functioning (e.g., increased propensity for violence [1]), particularly in those with substance use disorders (SUDs) and concurrent cognitive dysfunction [2,3]. Long-term substance use leads to physical and psychological dependence, profoundly affecting brain structure and function, resulting in CD [4,5,6]. Research shows that substances like alcohol, opioids, cocaine, and cannabis are associated with significant impairments in memory, attention, and executive function, primarily due to decreased frontal cortex activity, especially in the dorsolateral prefrontal cortex (DLPFC) [7,8,9]. For patients with SUDs, CD complicates withdrawal and recovery and increases relapse risk [10].

Current treatments for SUD-related CD rely on pharmacotherapy and psychotherapy. Pharmacotherapy often involves antipsychotics, antidepressants, and cognitive enhancers. Despite some evidence of efficacy, these treatments are frequently associated with limited success and notable side effects. Research indicates that relapse rates in drug dependence remain high, ranging from 40% to 60% [11]. Additionally, the side effects of pharmacotherapy can negatively impact cognitive functions, leading to impairments in working memory, cognitive flexibility, and overall executive function, as well as physical side effects like tachycardia and nausea [12]. Psychotherapeutic approaches, such as cognitive behavioral therapy and motivational interviewing, have shown some effectiveness, but their durability and generalizability require further research [13,14]. These treatments often require long-term interventions, imposing significant demands on both patients and healthcare systems. Furthermore, the effectiveness of psychotherapy is largely constrained by patient engagement and the availability of trained therapists. These limitations highlight the need for new, effective treatments that offer better accessibility and fewer side effects.

Transcranial direct current stimulation (tDCS) is a non-invasive brain stimulation technique that modulates neural activity in the cerebral cortex by applying low-intensity direct current through the scalp [15]. Due to its simplicity, low cost, and minimal side effects, tDCS has gained attention in SUD-related CD treatment research [16]. Unlike pharmacotherapy, tDCS does not involve the systemic administration of drugs, thereby avoiding many of the associated side effects. Moreover, tDCS sessions are relatively short and can be easily administered, potentially reducing the burden on healthcare systems and increasing patient compliance compared with long-term psychotherapeutic interventions [17]. Furthermore, studies indicate that tDCS can improve memory, attention, and executive function through mechanisms such as changes in resting membrane potential, cerebral blood flow, synaptic plasticity, neurotransmitter levels, and brain network connectivity [18,19]. Specifically, tDCS can modulate the default mode network by intervening in DLPFC excitability, activating executive control and salience networks, thereby affecting SUD cravings [20]. However, research on the application of tDCS in cognitive dysfunction in patients with SUDs remains limited.

Despite the growing interest in tDCS, there remains a lack of comprehensive understanding of its effectiveness and optimal application in treating cognitive dysfunction in individuals with SUDs. Existing studies are varied in their methodologies, target brain areas, stimulation parameters, and cognitive measures, leading to inconsistent results and unclear conclusions about the efficacy of tDCS. This systematic review evaluates the existing literature to assess the effectiveness of tDCS on cognitive dysfunction in individuals with SUDs, providing a basis for clinical practice and future research. We aimed to determine whether tDCS significantly improves cognitive function in SUD patients and identify optimal stimulation parameters. By systematically reviewing existing studies, we hope to promote the clinical application of tDCS in treating SUD-related CD and guide future research in this field.

## 2. Materials and Methods

### 2.1. Study Design

A systematic literature review was conducted according to the Preferred Reporting Items for Systematic Reviews and Meta-Analyses (PRISMA) guidelines [21]. Details of the review protocol were registered on the PROSPERO International Prospective Register of Systematic Reviews in April 2024 (http://www.crd.york.ac.uk/prospero/ (accessed on 14 April 2024), registration number: CRD42024531033). A meta-analysis was not conducted due to the high measurement and methodological heterogeneity in the selected studies.

### 2.2. Eligibility Criteria

Using the PICOS framework [22], studies were included based on the following criteria—population (P): studies of SUDs, including dependencies on alcohol, nicotine, cocaine, methamphetamine, opioids, or cannabis, based on standardized criteria (e.g., DSM-IV or DSM-Ⅴ), recruiting participants aged 18 years and older who exhibited cognitive dysfunction related to SUDs; intervention (I): studies that employed tDCS or high-definition transcranial direct current stimulation (HD-tDCS) as the sole intervention; comparison (C): studies that included either sham stimulation, a control group receiving no intervention, or an active control arm; outcome (O): studies that investigated substance-related cognitive function outcomes (such as attention, risk-taking, executive function, cognitive bias, and cognitive control) as primary or secondary outcomes using a validated measurement tool [e.g., frontal assessment battery (FAB)]; study design (S): studies that utilized either a parallel (between-subject) or crossover (within-subject) randomized controlled trial (RCT) design. With regard to outcome measures, we included studies that used cognitive–behavioral test tasks as well as those that employed cognitively relevant components (e.g., P3) measured by cognitively relevant tools (e.g., EEG).

Studies were excluded if they met any of the following criteria: (1) recruited participants who did not have SUDs or were not diagnosed using standardized criteria; (2) did not have a well-defined control group (for tDCS studies); (3) were not published in English; (4) included subjects with SUDs who also had other concurrent conditions (e.g., schizophrenia, Parkinson’s disease); or (5) were literature reviews, meta-analyses, dissertations, abstracts, conference presentations, or case reports.

### 2.3. Systematic Review Protocol

The titles and abstracts of studies identified through the search were independently screened for eligibility. Any discrepancies in judgment regarding eligibility were discussed until consensus was reached. Subsequently, all selected papers were read in full to check for all inclusion criteria. In cases where the search revealed duplicate publications, only one was included in the review.

### 2.4. Search Strategy

Before starting the current review, we searched for ongoing studies with the same scope in the WHO International Clinical Trials Registry Platform (WHO ICTRP) and the ClinicalTrials.gov databases but found no ongoing studies. Original articles were searched using the Ovid MEDLINE, PubMed, Web of Science, Embase, Scopus, and PsycINFO databases. The search was conducted in March 2024, using the keywords (“tDCS” OR “transcranial direct current stimulation”) AND (“substance use disorders” OR “addiction”) AND (“cognitive function” OR “risk-taking” OR “executive function”). The search terms are shown in Table 1. Additionally, references from the retrieved literature were scanned to identify any further relevant studies.

### 2.5. Data Sources, Study Selection, and Data Extraction

The following data were independently extracted from the included papers: substance and clinical status (whether in treatment or not), number of participants in the substance disorder group and their gender distribution, mean age and standard deviation, follow-up visits, the task used to assess cognitive function, outcomes defining cognitive function, the use of tDCS and the stimulation site, stimulation parameters, number of stimulation sessions, and the results of any active vs. sham comparison.

In our systematic review, we utilized the Cochrane risk-of-bias assessment tool to evaluate the quality of the included studies [23]. By providing a systematic and standardized approach to evaluate the potential biases in study design, conduct, and reporting, the Cochrane risk-ofbias assessment tool ensured the clarity and validity of our findings. Each component was rated as having a “high”, “low”, or “unclear” risk of bias. We utilized Review Manager 5.4.1 software (Cochrane Collaboration, Oxford, UK) for the analysis.

## 3. Results

### 3.1. Search Results and Study Selection

**Figure 1:**
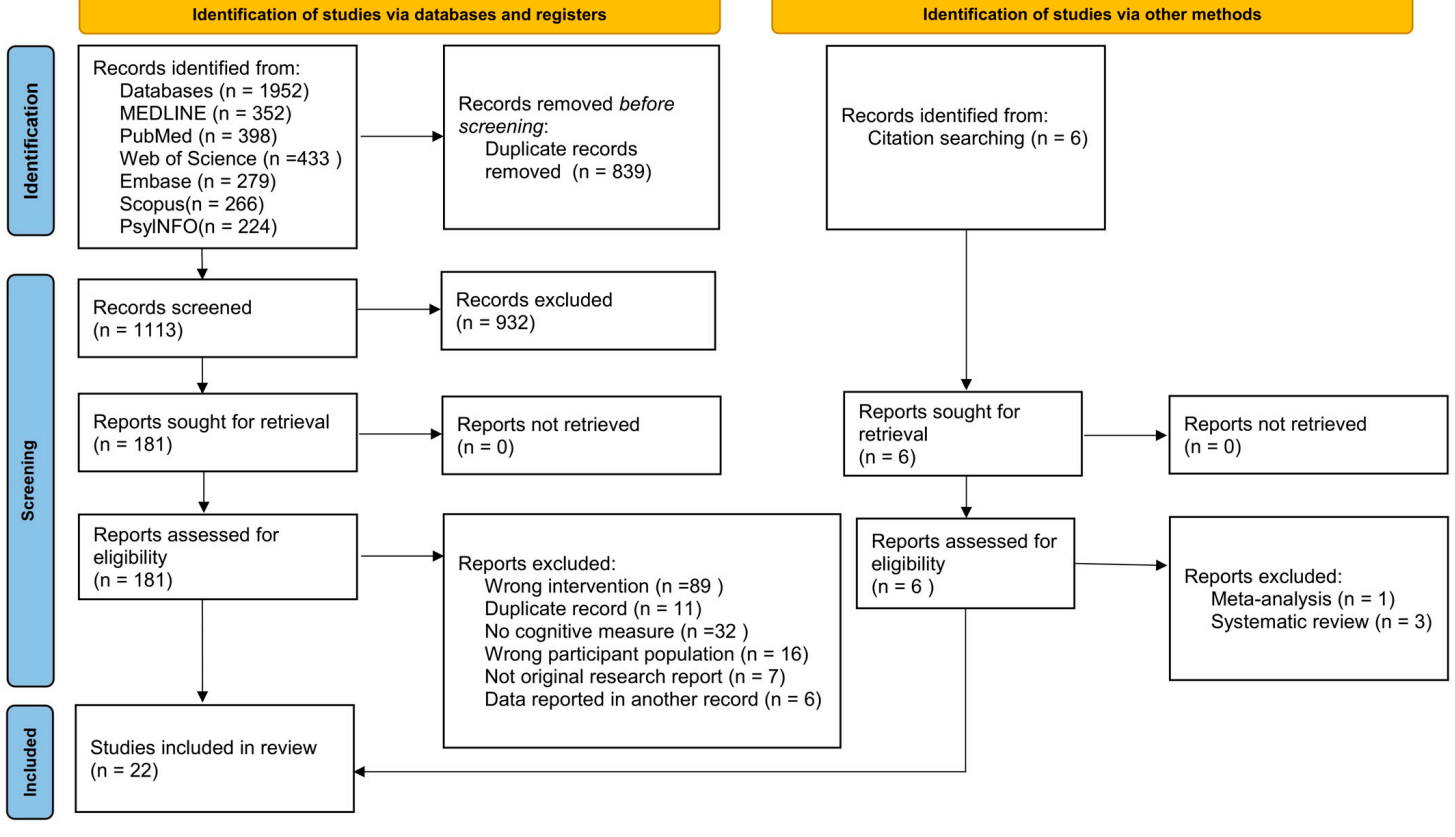
PRISMA flowchart diagram. Modified from Page et al. [21]. For more information, visit http://www.prisma-statement.org/ (accessed on 15 April 2024).

In total, 1958 articles were identified in the systematic literature search (MEDLINE: 352; PubMed: 398; Web of Science: 433; Embase: 279; Scopus: 266; PsycINFO: 224; other sources: 6). After removing duplicate articles, 1113 articles were screened based on their titles and abstracts, resulting in the exclusion of 932 articles. Following a full-text assessment, 163 records were further excluded due to reasons such as wrong interventions (*n* = 89), duplicate records (*n* = 11), lack of cognitive measures (*n* = 32), wrong participant population (*n* = 16), not being original research reports (*n* = 7), and data being reported in another record (*n* = 6). Two studies were included in this research after excluding meta-analysis (*n* = 1) and systematic reviews (*n* = 3) from the citation searching. Ultimately, 22 articles were deemed eligible for the systematic review. The literature search process is illustrated in Figure 1.

### 3.2. Quality and Risk-of-Bias Assessment

**Figure 2:**
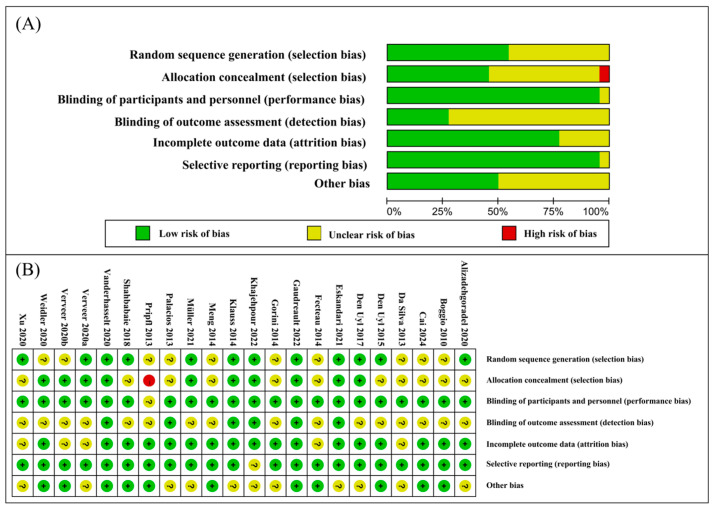
Quality and risk-of-bias assessment. Risk-of-bias graph (**A**): review authors’ judgments about each risk-of-bias item presented as percentages across all included studies; and risk-of-bias summary (**B**): review authors’ judgments about each risk-of-bias item for each included study (red, green and yellow circles indicate "high", "low" and "unclear" risk of bias, respectively).

The overall quality of the included studies was high, with all studies employing a sham-controlled design and assessing cognitive function immediately following the tDCS intervention. However, 14 of the studies exhibited an unclear risk of selection bias due to ambiguous random sequence generation in 10 articles and unclear allocation concealment in 11 articles. One study demonstrated a high risk of bias. One study showed unclear blinding of participants and personnel. Sixteen studies had an unclear risk regarding the blinding of outcome assessments, and one study had an unclear risk of selective reporting. Eleven studies presented an unclear risk of other biases. The risk-of-bias graphs and a summary are presented in Figure 2.

### 3.3. Study Characteristics

An overview of the literature characteristics is given in Table 2. All studies used a sham-controlled design. In total, 770 SUDs were included in 22 studies, with 612 men (79.48%) and 158 women (20.51%). Alcohol-dependent patients in one of these studies were excluded from these SUDs because they were using medications affecting the central nervous system to treat other psychiatric disorders, such as depression and post-traumatic stress disorder, during the experimental period [24]. The mean ages of the participants ranged from 21.24 ± 1.35 years [25] to 48.80 ± 8.90 years [26]. All studies used sham tDCS as a control condition. The studies covered methamphetamine (three papers), marijuana (one paper), alcohol (six papers), opioids (one paper), nicotine (seven papers), and cocaine (three papers). The substance used was unknown in one of the conducted studies [27]. Regarding the tDCS application protocol, 18 studies utilized the offline mode (post-stimulation testing), while 4 studies employed the online stimulation mode (testing during stimulation) [28,29,30,31].

### 3.4. Summary of Findings

In 21 studies, the intervening brain region for anodal tDCS (a-tDCS) was the DLPFC (54.16% were left-sided), and three of these studies included both left- and right-sided stimulation protocols [28,29,36]. Interestingly, the studies that produced a significant effect of a-tDCS over DLPFC on the left and right side [25,27,32,34,35,36,37,45] were the same in number, eight, accounting for 38.09% of all studies that targeted DLPFC. Also, one of the studies included IFG [43]. Moreover, one study included intervention in the frontal–parietal–temporal association area (FPT) of the brain [41]. There were twenty-one studies applying a cephalic montage of the return electrode [24,26,27,28,29,30,31,32,33,34,35,36,37,38,39,40,41,42,43,44], and three studies applied an extracephalic montage of the return electrode [25,34,45], including one study that used two electrode montages [34]. The tDCS intervention sessions ranged from 1–15 sessions (1 sessions [24,25,26,27,28,29,33,34,36,40,41,43], 3 sessions [38], 4 sessions [30], 5 sessions [31,39,42,44,45], 10 sessions [32,37], and 15 sessions [35]), and three studies involved follow-up tests 1–3 months after the immediate intervention [31,32,38]. Four of the ten multi-session intervention studies reported a significant effect [32,37,39,45], and five of the twelve single-session intervention studies showed an effect [26,29,33,34,36]. Only one study used HD-tDCS interventions [27]. The tDCS intensities chosen for the studies included 0.45 mA (1 papers) [29], 1 mA (3 papers) [25,41,43], 1.5 mA (2 papers) [24,36], and 2 mA (16 papers). The tDCS sham-stimulation protocols included (1) switching off the current after the first 20–30 s of application (15 papers), (2) switching off at the beginning and end of the 30–60 s of application (5 papers) [29,33,35,39,40], (3) varying the current from 0.3 mA to 2.0 mA and back again at a rate of 0.1 mA per second (1 paper) [42], and (4) varying the current from 0 mA to 2.0 mA and back again for the first and last 60 s (1 paper) [33]. One study did not report sham-stimulation protocols [37].

The tDCS densities chosen for the studies included 0.029 mA/cm^2^ (2 papers) [25,43], 0.047 mA/cm^2^ (1 paper) [36], 0.057 mA/cm^2^ (14 papers), 0.08 mA/cm^2^ (1 paper) [42], 0.085 mA/cm^2^ (1 paper) [29], and 0.637 mA/cm^2^ (1 paper) [33], and in 2 papers these values were not reported. The tDCS durations chosen were 10 min (2 papers) [25,43], 13 min (1 paper) [44], 15 min (3 papers) [27,28,29], 20 min (12 papers), and 30 min (1 paper) [39], and 3 papers adopted two 13 min interventions with a 20 min break in between [31,34,38].

The included articles reported the effects of tDCS on participants’ performance across a wide range of cognitive tasks, which we categorized into four domains: (1) executive function; (2) attention and alertness; (3) risk-taking; and (4) other cognitive functions (impulsivity and self-control; motivation and willingness; reactive aggression; basic response time). Some studies addressed the effect of multiple stimulation protocols (for example, left as well as right anodal tDCS) (eight papers) or of multiple outcome measures [for example, multiple tasks (eight papers) or one task with multiple outcome measures (eight papers)] or a combination of these two factors (seven papers). There was high variability between studies concerning the stimulation protocols applied. 

#### 3.4.1. Executive Function

Eleven studies addressed executive function, including two on working memory, two on cognitive flexibility, six on inhibitory control, and three on overall executive function [24,25,26,27,31,32,38,39,42,44,45]. Two of these studies examined cognitive flexibility and inhibitory control simultaneously [42], while one study investigated working memory, cognitive flexibility, and inhibitory control concurrently [32]. A summary of findings on executive function is illustrated in Table 3. For working memory, one study used the N-back task [32]. The other study employed a working memory/attention task, where participants were asked to maintain visual or audiovisual information in their memory while performing a search task [27]. These studies assessed three outcome measures in total. One study indicated that a-tDCS over the left dorsolateral prefrontal cortex (DLPFC) improved working memory performance on the N-back task compared to pre-intervention and the sham group, with effects observed both immediately and during follow-up [32]. However, there was no significant difference between the active and sham groups in Cai’s study [27].

For cognitive flexibility, one study used Wisconsin Card Sorting Task (WCST), revealing differences in set-shifting abilities post-intervention [32]. The other study used Dimensional change card sort task (DCCS), which evaluates an individual’s ability to shift between different rules or dimensions of a task [42]. One study reported two outcome measures [32], and the other study did not report an outcome measure [42]. One study indicated that a-tDCS over the left DLPFC improved cognitive flexibility performance in the WCST task compared with pre-intervention and the sham group, with effects observed both immediately and during follow-up [32]. However, there was no significant difference between the active and sham groups in Müller’s study [27].

For inhibitory control, four studies used the go/no-go task, which measures an individual’s ability to suppress a response to certain stimuli while responding to others [31,32,38,42]. One of the studies used the stop-signal task (SST) in addition to the go/no-go task. One of the studies employed a reward go/no-go task to assess how monetary incentives affected participants’ response tendencies, thereby underscoring the interaction between reward processing and cognitive control mechanisms [26], and meanwhile used ERPs to collect N2, N3, and ERN amplitude changes during the go/no-go task; these ERP metrics were associated with conflict detection, inhibitory control, sustained attention, error detection, and error correction. Another study used the ultimatum game to assess the impact of rewards on participants [39]. In addition, another study used the stop-signal reaction-time task (SSRT), similar to the go/no-go task [24]. 

The studies assessed seven outcome measures in total. One study did not report its outcome measures [42]. One study indicated that a-tDCS over the left DLPFC improved inhibitory control performance on the go/no-go task compared with pre-intervention and the sham group, with effects observed both immediately and during follow-up [32]). Another study showed that a-tDCS over the right DLPFC reduced go-ACC in the reward condition compared with the sham group, while no effect was observed on no-go-ACC [26]. One study found that a-tDCS over the right DLPFC did not yield a beneficial effect in the immediate post-stimulation period but did result in a decrease in no-go-response time (RT) and a reduction in no-go-N3 amplitude compared with the sham group at a three- month follow-up [38]. Two studies applied a-tDCS over the left/right DLPFC in the go/no-go task, respectively, and did not find a significant effect on the go/no-go results [31,42]. One study indicated that a-tDCS over the right DLPFC improved inhibitory control performance in the cigarettes ultimatum game compared with the sham group [39]. In addition, one study indicated that a-tDCS over the right DLPFC improved inhibitory control performance in the SSRT task compared with the sham group [24]. 

To investigate overall executive function, three studies used frontal assessment battery (FAB), which assesses various executive functions to evaluate an individual’s prefrontal cognitive abilities [25,44,45]. Two studies indicated that a-tDCS over the left DLPFC improved executive function performance in the FAB compared with the sham [25,45]. One of these found this effect in individuals classified as Lesch Ⅳ (most severe alcohol use) only [25]. In addition, one study indicated that a-tDCS over the right DLPFC had no significant effect on FAB performance [44].

#### 3.4.2. Attention and Alertness

Eight studies addressed attention and alertness, including three on sustained attention and alertness, two on attentional bias, and three on motivated attention [25,27,33,34,40,41,42,45]. A summary of the findings on attention and alertness is illustrated in Table 4. For sustained attention and alertness, one study used the visual attention task, which assesses an individual’s ability to maintain attention and identify targets under varying load conditions [40]. One study used the continuous performance task (CPT), which measures a person’s sustained and selective attention and impulsivity by requiring them to respond to specific target stimuli while ignoring non-target stimuli [42], and another study used a WM/attention task [27]. These studies assessed four outcome measures in total. One of these studies did not report its outcome measures [42]. Two studies indicated that a-tDCS over the left DLPFC was associated with no significant difference between the active and sham groups [40,42]. Cai et al. found that HD-tDCS over the left DLPFC decreased attention to RT in the WM/attention task compared with other groups [27].

To measure attentional bias, one study used an attention task that assessed how participants allocated their attention to specific visual stimuli by recording their eye movements with an eye-tracking system [41]. Another one study used a probe detection task (PDT), which evaluated attentional bias by measuring reaction times to probes replacing drug-related versus neutral cues on a screen [34]. These studies assessed four outcome measures in total. Meng et al. indicated that double-cathodal tDCS (c-tDCS) over the bilateral FPT led to a declining trend in fixation counts of smoking-related cues, but the results were not significantly different from the sham [41]. Another study showed that a-tDCS over the left DLPFC and c-tDCS over the right shoulder/DLPFC decreased attentional bias compared with the sham group [34].

Regarding motivated attention, three studies captured event-related potentials (ERPs) elicited by substance cues and determined the effect of tDCS on levels of attentional resources and motivated attention by analyzing correlated amplitude changes. These studies assessed two outcome measures in total. One study showed that a-tDCS placed on the left DLPFC decreased the P3 amplitude compared with the sham [25]. One study found that a-tDCS placed on the right DLPFC decreased P3 amplitude but had no effect on LPP amplitude compared with the sham group [33]. In addition, one study indicated that a-tDCS over the left DLPFC increased P3 amplitude particularly in Lesch IV (severe alcohol use) participants compared with the sham, but decreased P3 amplitude in Lesch II (mild alcohol use) subjects compared with the sham [25].

#### 3.4.3. Risk Taking

Six studies addressed risk taking [28,29,31,32,36,39]. A summary of findings on risk taking is illustrated in Table 5. Each of the six studies was tested using six risk-taking tasks, including the following: (1) risk task: participants chose between low-risk/low-reward and high-risk/high-reward options, to assess their risk-taking propensity and decision-making strategies [28]; (2) Columbia card task (CCT) [29]; (3) balloon analogue risk task (BART) [32,36]; (4) risky decision-making paradigm [39]; (5) game-of-dice task (GDT) [36]; (6) two-choice gambling task (TCGT) [31]. These studies assessed eleven outcome measures in total. One study indicated a-tDCS over the DLPFC increased risk-taking in the risk task [28]. One study indicated a-tDCS over the left/right DLPFC decreased risk-taking in the CCT compared with the sham [29]. Two studies indicated a-tDCS over the left/right DLPFC decreased risk-taking in the BART compared with the sham [32,36]. Meanwhile, Gorini et al. showed that a-tDCS over the right DLPFC decreased risk-taking in the GDT, but a-tDCS over the left DLPFC increased risk-taking compared with the sham. In addition, two studies indicated that a-tDCS over the right DLPFC had no significant effect on the risk task or the TCGT compared with the sham [31,39].

#### 3.4.4. Other Cognitive Functions

There were six studies on other cognitive functions [24,30,35,37,42,43]. Three of these studies focused on impulsivity and self-control [35,37,42], two on motivation and willingness [30,43], and one study addressed impulsivity and self-control along with motivation and willingness [35]. Additionally, one study examined reactive aggression [24], and another investigated basic response time in relation to impulsivity [42]. A summary of findings on executive function is illustrated in Table 6. To assess impulsivity and self-control, two studies used delay-discounting tasks (DDTs) to measure individuals’ tendency to prefer smaller, immediate rewards over larger, delayed rewards, a concept known as delay discounting [35,42]. One of these used the Kirby delay-discounting task (KDDT), which is similar to the DDT [35]. One study used the Barratt impulsiveness scale version 11 (BIS-11), which measures an individual’s impulsivity when maintaining attention, planning actions, and responding impulsively [37]. These studies assessed two outcome measures in total. Müller et al. did not report their outcome measures [42]. One study indicated that a-tDCS over left DLPFC decreased impulsivity performance on BIS-11 compared with the sham [37]. One study indicated that a-tDCS over the right DLPFC improved self-control performance in the KDDT compared with the baseline test [35]. In addition, another study indicated that a-tDCS over the left DLPFC had no significance effect compared with the sham [42].

To assess motivation and willingness, three studies included testing using three tasks, including (1) the affective implicit association test (affective IAT), measuring implicit associations between alcohol and affective attributes such as positive and negative words [43]; (2) the contemplation ladder (CL) task, evaluating participants’ readiness to change their drug use behavior [35]; (3) the alcohol approach-avoidance task (AAT), measuring automatic approach and avoidance tendencies towards alcohol-related stimuli [30].

For assessment of reactive aggression, Weidler et al. used the modified Taylor aggression paradigm (mTAP) [24]. This method measures aggression and response inhibition by having participants choose a punishment level for an opponent and then engage in a reaction-time task to determine whether they can administer the punishment. This study indicated that a-tDCS over the right DLPFC had no significance effect on mTAP compared with the sham. Regarding basic response time, Müller et al. indicated that a-tDCS over the left DPFC had no significant effect on SRTT compared with the sham [42].

## 4. Discussion

The primary aim of this systematic review was to summarize the current evidence on the therapeutic effects of tDCS for CD in SUDs. Additionally, we aimed to identify the most effective tDCS protocols for improving cognitive function in SUDs. To achieve these goals, we manually screened scientific papers from six databases and reported their characteristics based on the stimulation protocols used in the studies. Our review identified a total of 22 original studies on SUDs relating to methamphetamine, marijuana, alcohol, opioids, nicotine, and cocaine. The reviewed studies demonstrated the potential efficacy of tDCS in improving executive function, risk-taking, and attention in SUDs, but many differences between studies were also highlighted. Our study identified a burgeoning number of reports of positive effects of tDCS on modulating cognitive function in SUDs. A-tDCS over the DLPFC has been associated with improved executive function [24,25,26,32,39,45], enhanced attention [27,33,34,45], reduced risk-taking [29,32,36], decreased impulsivity in opioid use disorders [37], enhanced emotional processing capabilities [43], and enhanced motivation for drug withdrawal [35]. Importantly, we also found some studies in which tDCS had no significant effect on executive function [27,31,38,42,44], attention function [25,40,41,42], risk-taking [31,39], impulsivity and self-control [35,42], alcohol approach bias [30], reactive aggression [24], or simple response time [42], and there were two reports of increased risk-taking [28,36].

The variability in findings is likely to have been due to the diverse range of targeted brain areas, stimulation parameters, cognitive measures, and populations examined in the reviewed studies. In the following sections, we explore how these factors may have influenced the outcomes, offering insights that could guide future research on the therapeutic potential of tDCS for SUDs.

### 4.1. Target Brain Area

A review of previous studies revealed that the DLPFC was selected as the target area for stimulation in almost all studies. This suggests that tDCS modulation of DLPFC excitability is instrumental in modulating cognitive function in SUDs. This may be due to the fact that the DLPFC is an important region in the cognitive control network associated with substance abuse-induced dysfunction in decision making and self-control [8,46,47]. Previous studies investigating decision-making processes in SUDs found that the left DLPFC mediates reward-based motivation, whereas the right DLPFC is involved in withdrawal-related behaviors and inhibition [48]. The current study found that more studies placed a-tDCS over the left DLPFC (54.16%), but the number of studies that produced significant effects was the same for both the left and right DLPFC. Moreover, a-tDCS over the left DLPFC had as many stimulus sessions (≥5 sessions) as a-tDCS over the right DLPFC (≥5 sessions). This finding contradicts the conclusions of a recently published systematic review, which suggested that right-sided A-tDCS appeared to be most effective for SUDs [49]. Nevertheless, Gorini et al. found that a session of a-tDCS stimulation of the left DLPFC increased risk-taking behavior in cocaine SUDs, but a-tDCS stimulation of the right DLPFC increased safe behavior [36]. This suggests that tDCS modulation of right DLPFC excitability is instrumental in enhancing cognitive function in SUDs.

Furthermore, the vast majority of studies used a bi-hemispheric frontal stimulation montage, i.e., a-tDCS-F3/c-tDCS-F4, which has been shown to be effective in improving cognition and reducing cravings in SUDs [32,50]. However, some studies showed null or even opposite effects [28,39,51,52]. This montage may have led to difficulties in interpreting the results, i.e., whether it was the increase in left DLPFC excitability caused by the a-tDCS or the inhibition of right DLPFC excitability caused by the c-tDCS that influenced the results. Three studies used an extracerebral electrode protocol, with a-tDCS placed on the left DLPFC and a reference electrode placed on the contralateral deltoid or shoulder, and two of these studies reported positive results [34,45]. In addition to DLPFC, two other studies used a-tDCS over the IFG and FPT as target brain regions, but neither had significant effects [41,43]. 

However, there were still other studies that did not find a significant effect of a-tDCS placed over the DLPFC. One study even found that a-tDCS over the DLFPC increased risk-taking tendencies in marijuana addicts [28]. This may indicate that excessive excitability is potentially counterproductive to improving cognitive functioning in SUDs. Furthermore, this may be related to the localization of brain regions by the 10–20 EEG system typically used in research, which ignores individual differences in brain morphology and network structure. Therefore, the future use of neuronavigation guidance for personalized localization of brain regions is necessary to improve the consistency of these findings. 

### 4.2. Stimulation Parameters

Variability in treatment effects in tDCS studies may have been due to differences in stimulus parameters, such as frequency, intensity, density, and treatment duration. As shown in Table 3, Table 4, Table 5 and Table 6, the studies reviewed here employed various stimulation parameters. The findings suggest that an optimal stimulation protocol may involve a current of 2 mA, continuous stimulation for 20 min, and a current density of 0.057 mA/cm^2^ [26,32,33,35,37,45]. This may be due to the high number of stimulation sessions (≥5 sessions) in these protocols. This result aligns with studies on tDCS in patients with mild cognitive impairment (MCI), which also found that higher numbers of stimulation sessions (≥5 sessions) were beneficial. Given that both SUDs and MCI involve significant cognitive dysfunction and that tDCS has shown promise in treating these impairments, it is valuable to compare the protocols and outcomes across these conditions [53,54]. Recently, a meta-analysis indicated that tDCS treatment involving more than 10 sessions and current densities exceeding 2.5 mA/cm^2^ was most effective in improving cognitive dysfunction in MCI patients [55]. However, the current study found that 5 of the 12 single-session intervention studies were effective [26,29,33,34,36], which was similar to the number of effective multi-session intervention studies [32,37,39,45]. This implies that the mechanisms of influence in acute and long-term interventions need to be further explored in future research.

Regarding current intensity, most studies used intensities greater than or equal to 1.5 mA. The effect of tDCS, which delivers current to the targeted brain region via electrode pads, may be influenced by cranial anatomical features, such as cranial thickness and morphology [56]. Meta-analysis likewise showed that a-tDCS significantly reduced cravings with at least 1.5 mA of current intensity [57].

Regarding current density, almost all effective protocols used a current density of at least 0.057 mA/cm^2^. This is likely to have been due to the fact that larger electrodes reduce the focusing effect, thereby impacting a broader range of brain regions [58]. However, one study achieved a current density of 0.085 mA/cm^2^ with a current intensity of 0.45 mA by using smaller electrode [29]. This small anode/large cathode protocol minimizes cathodic effects while enhancing anodic resolution. Additionally, Cai et al. reported positive outcomes using a more focused HD-tDCS, suggesting that higher-resolution tDCS montages may improve efficacy [27]. 

Regarding stimulus duration, most of the effective protocols used a duration of at least 15 min. Previous studies have demonstrated that the effects of tDCS are time-dependent. For instance, a 9 min tDCS intervention produced sustained effects for up to 1 h, whereas a 35-min intervention produced effects lasting less than 5 min [59]. However, some studies have shown positive outcomes with longer durations. Fecteau et al. found that applying 2 mA tDCS over the DLPFC for 30 min improved inhibitory control in smoking-related SUDs [39]. Similarly, Shahbabaie et al. reported that 2 mA tDCS with two 13-min sessions separated by a 20-min interval improved attentional bias in methamphetamine-associated SUDs [34]. A recent meta-analysis on the efficacy of tDCS interventions in SUDs aligns with these findings, demonstrating that treatment was effective when the duration of a-tDCS stimulation was 20 min, the current intensity exceeded 1.5 mA, and the current density was greater than 0.042 mA/cm^2^ [57]. These results suggest that while the effects of tDCS can be time-dependent, certain durations and intensities can enhance its efficacy in treating SUDs. Nonetheless, other studies using the same protocol failed to find a significant effect on improving cognitive function, indicating that further exploration of the optimal tDCS protocol for improving cognitive function in SUDs is necessary.

### 4.3. Task Requirements and Cognitive Measures

Some of the differences between studies may have been due to the use of different tasks within the same domain. Executive function involved nine tests, attention and alertness involved six tests, risk-taking involved six tests, impulsivity involved three tests, and motivation and willingness involved three tests. For example, it was found that the effect of tDCS on risk-taking performance varied depending on the specific task performed (BART/GDT) [36]. On the one hand, when measuring the intervention effects of tDCS, tasks of different levels of difficulty within the same cognitive domain produced different results [60]. On the other hand, different tasks may reflect different degrees of cognitive functioning, which may modulate the excitability of participating brain regions [61]. Since tDCS modulates subthreshold excitability, such changes may affect the regulation of brain region excitability [62,63]. In addition, the extent to which a task involves reward or loss may affect task-related neural processing and thus, the effects of tDCS [64]. Reward or failure stimulates the release of dopamine, which may influence the effects of tDCS [65,66].

The present study also reported the online/offline mode of the test tasks used in different studies [67,68], with only four studies using the online mode, of which only one had a significant effect [29]. Given the variability in task types and their impact on tDCS efficacy, future research should focus on standardizing the tasks used within each cognitive domain.

### 4.4. Inter-Individual Variation

To be included in the review, studies needed to have involved participants with an addiction to a substance. Consequently, there were variations between studies in terms of substance type (e.g., methamphetamine, marijuana, alcohol, nicotine), severity of addiction, and participant status. These variations may have affected the effectiveness of tDCS, as inter-individual differences play a significant role [62]. The effects of tDCS are influenced by structural factors, brain state, and the dopaminergic system. Neural differences associated with addiction can significantly affect responsiveness to tDCS [69]. This variability was evident in the follow-up studies: only three studies conducted follow-up investigations, with two finding significant effects of tDCS at one [32] and three [38] months after the last stimulation, respectively. The variation in results may be related to the type of substance addiction and participant status, as studies without significant effects involved clinically studied cocaine SUDs [31].

Baseline cognitive function may also vary among SUDs of different substance types. For example, Young et al. found differences in motor timing ability between alcohol-dependent and drug-dependent patients after rehabilitation [70]. Baseline cognitive differences within the same substance type may also have influenced tDCS effects [71,72]. Furthermore, the age and gender of subjects can impact the effect of tDCS on cognitive performance interventions [73,74]. Our review found that SUDs were more common in men (79.48%) than in women (20.51%), which may characterize an imbalance in this group [75]. This disparity also exists across different ages and races [75]. 

Therefore, future research should focus on standardizing participant characteristics and considering demographic factors to better understand the efficacy of tDCS in treating SUDs.

## 5. Limitations and Future Reassurance

The studies included in this review exhibited significant heterogeneity in terms of the targeted brain areas, stimulation parameters, cognitive measures, and participant characteristics, complicating the synthesis of results and the determination of optimal tDCS protocols. Additionally, many of the studies had small sample sizes and short follow-up periods, limiting the generalizability and understanding of the long-term effects of tDCS on cognitive function in SUDs. Variability in tDCS protocols and differences in blinding and control conditions further challenge the validity of the findings. Moreover, this review examined only the effects of tDCS on cognitive function in SUDs. Recent studies have used a combination of tDCS and cognitive training to modulate cognitive function in SUDs [16].

Future research should aim to standardize tDCS protocols, ensuring consistency in terms of the brain areas targeted, stimulation parameters, cognitive measures, and participant characteristics. Studies should also include larger and more diverse samples with longer follow-up periods to improve the generalizability of the findings and enhance the understanding of the long-term effects of tDCS on cognitive function in SUDs. Additionally, investigating the underlying mechanisms of tDCS through fMRI studies [76], combining tDCS with cognitive training or other therapeutic interventions, and considering individualized approaches based on participant-specific factors will help optimize treatment outcomes. Enhanced blinding techniques and rigorous control conditions are essential in order to improve the validity and reliability of future studies. 

## 6. Conclusions

This systematic review provides evidence supporting the potential application of tDCS in treating CD related to SUDs. The findings suggest that tDCS can improve various cognitive functions, such as executive function, attention, and risk-taking behaviors. Effective tDCS protocols identified include a current intensity of 2 mA, continuous stimulation for 20 min, and a current density of 0.057 mA/cm^2^. Practically, tDCS presents a promising non-invasive, cost-effective, and low-risk intervention that can be integrated into existing treatment programs to enhance cognitive function in SUD patients. Future research should focus on standardizing protocols, conducting larger and diverse sample studies, implementing long-term follow-up assessments, investigating underlying neural mechanisms, exploring combination therapies, and developing individualized treatment plans. Enhanced blinding techniques and rigorous control conditions are essential to improve the validity and reliability of findings. This review underscores the potential role of tDCS in enhancing cognitive function in SUD patients and emphasizes the need for more consistent and targeted research in this field.

## Figures and Tables

**Table 1:** Search terms used to identify relevant articles in the databases.

1	alcohol	16	opiate
2	alcoholic	17	opioid
3	cannabis	18	heroin
4	cigarette	19	benzodiazepine
5	crack	20	addiction
6	cocaine	21	substance
7	stimulant	22	drug
8	smoking	23	“substance use disorder”
9	sedative	24	attention
10	tobacco	25	executive function
11	hallucinogen	26	cognitive bias
12	amphetamine	27	risk taking
13	methamphetamine	28	cognitive control
14	marijuana	29	“transcranial direct current stimulation”
15	nicotine	30	“tDCS”
30	Substance use search terms: 1 OR 2 OR 3 OR 4 OR 5 OR 6 OR 7 OR 8 OR 9 OR 10 OR 11 OR 12 OR 13 OR 14 OR 15 OR 16 OR 17 OR 18 OR 19 OR 20 OR 21 OR 22 OR 23		
31	Cognitive dysfunction terms: 24 OR 25 OR 26 OR 27 OR 28		
32	Neuromodulation search terms: 29 OR 30		
33	Final search terms: 30 AND 31 AND 32		

**Table 2:** Characteristics of the studies.

Reference	Substance	Sample					# Ss	Follow-Up	Study Design
		Degree	Stage	Medication	Size (M–F)	Age [Mean (SD)]			
Alizadehgoradel et al., 2020 [32]	Methamphetamine	≥1 year	Withdrawal	NR	39 (39–0)	34.83 (9.16)	10	1 month later	DB, RPG, and SC design
Khajehpour et al., 2022 [33]	Methamphetamine	NR	Withdrawal	Yes	42 (42–0)	32.7 (1.6)	1	No	DB, R, and SC design
Shahbabaie et al., 2018 [34]	Methamphetamine	1 year of withdrawal, at least 3 times a week for the last month before withdrawal	Withdrawal	NR	90 (90–0)	30.76 (6.18)	1	No	DB, R, SC, and crossover design
Verveer et al., 2020b [31]	Cocaine (clinical)	Withdrawal ≥ 1 week	Withdrawal	Yes	59 (47–12)	39.72 (10.36)	5	3 months later	DB, R, and SC design
Gaudreault et al., 2022 [35]	Cocaine (clinical)	NR	Withdrawal	Yes	14 (12–2)	43.1 (11.87)	15	No	DB, R, and SC design
Gorini et al., 2014 [36]	Cocaine (clinical)	Mean dosing history 12.63 ± 6.4 years	Withdrawal	No	18 (10–8)	38.4 (8.2)	1	No	SB, R, and SC design
Eskandari et al., 2021 [37]	Opioid (clinical)	Mean dosing history = 12.26 years	Withdrawal	NR	31 (31–0)	33.16 (8.84)	10	No	DB, R, and SC design
Boggio et al., 2010 [28]	Marijuana	Mean dosing history 5.8 ± 2.7 years, 5.5 ± 1.9 times per week	Non-withdrawal	No	25 (15–10)	22.8 (2.6)	1	No	DB, R, SC, and Single-center design
Cai et al., 2024 [27]	NR	NR	Withdrawal	NR	25 (25–0)	32.18 (2.45)	1	No	R, SC, repeated-measures, and crossover design
Verveer et al., 2020a [38]	Nicotine	FTND mean scores = 3.4, 11.2 cigarettes per day	Non-withdrawal	No	73 (37–36)	22.3 (4.7)	3	1 day and 3 months later	DB, R, SC, and between-subjects design
Fecteau et al., 2014 [39]	Nicotine	1 heavy smoker (25 cigarettes per day), 7 moderate smokers (15–24 cigarettes per day) and 4 light smokers (<15 cigarettes per day)	Non-withdrawal	NR	12 (5–7)	36.3	5	No	SB, R, SC, and crossover design
Weidler et al., 2020 [24]	Nicotine	≥10 cigarettes per day	NR	NR	17 (17–0)	41.47 (12.06)	1	No	DB, R, SC design
Xu et al., 2013 [40]	Nicotine	FTND mean scores = 5.7, 16.4 cigarettes per day	At least 10 h of withdrawal before intervention only	No	24 (21–3)	45 (7.6)	1	No	SB, SC, and counterbalanced design
Meng et al., 2014 [41]	Nicotine	Mean dosing history 6.6 ± 6.7 years, 15.8 ± 6.4 cigarettes per day	Non-withdrawal	No	27 (27–0)	23.7 (7.2)	1	No	SB, R, and SC design
Müller et al., 2021 [42]	Nicotine	10.74 ± 5.65 cigarettes per day	Non-withdrawal	No	44 (21–23)	29.70 (10.41)	5	No	SB, R, and SC design
Pripfl et al., 2013 [29]	Nicotine	Mean dosing history ≥ 1 year, ≥10 cigarettes per day	Withdrawal	No	18 (8–10)	22.4 (2.5)	1	No	SC, crossover design
Palacios et al., 2013 [25]	Alcohol	32.6% were Lesch type I, 14.3% type II, 28.6% type III, and 24.5% type IV	Withdrawal	Yes	49 (45–4)	48.8 (8.9)	1	No	SB, R, SC, and mixed-design
den Uyl et al., 2015 [43]	Alcohol	AUDIT scores > 8	NR	NR	41 (15–26)	21.7 (3.0)	1	No	SB, R, and SC design
Klauss et al., 2014 [44]	Alcohol (clinical)	Mean dosing 240 g per day	Withdrawal	Yes	33 (32–1)	44.8 (8.3)	5	No	SB, R, SC, and mixed-design
da Silva et al., 2013 [45]	Alcohol	Lesch’s type IV alcohol-dependent	NR	NR	13 (13–0)	49 ##	5	No	SB, R, SC, and crossover design
Vanderhasselt et al., 2020 [26]	Alcohol	AUDIT scores ≥ 16	Withdrawal	NR	45 (30–15)	21.24 (1.35)	1	No	DB, R, SC, and crossover design
den Uyl et al., 2017 [30]	Alcohol	AUDIT mean scores = 24.3	Withdrawal	NR	31 (20–11)	46.8 (9.0)	4	No	DB, R, and SC design

Note. AUDIT = alcohol use disorders identification test; M = male; FTND = Fagerström test for nicotine dependence; F = female; NR = not reported; # = numbers; R = randomized; RPG = randomized parallel group; Ss = sessions; SB = single-blind; SC = sham-controlled; SD = standard deviation; DB = double-blind; ## = median age per group.

**Table 3:** Summary of findings on executive function.

ID	Protocol							Assessment		Results
	T	Online/Offline	Area	EP	Intensity	Duration	Density	Task and Measures	Indicators	
Working memory										
Alizadehgoradel et al., 2020 [32]	tDCS	Offline	DLPFC	A-F3/C-F4 in A and S	2 mA (sham A)	20 min	0.057 mA/cm^2^	N-back	ACC RT	CWG: ACC ↑ and RT ↓ after active tDCS intervention and follow-up compared with pre-intervention; CBG: ACC ↑ and RT ↓ at post-intervention and follow-up for active tDCS compared with sham group.
Cai et al., 2024 [27]	HD-tDCS	Offline	DLPFC	CE (either A/C)-F3, RE-AF3, F1, F5, and FC3 in A and S	2 mA (sham A)	15 min	NR	WM/Attention task	MACC	NS
Cognitive flexibility										
Alizadehgoradel et al., 2020 [32]	tDCS	Offline	DLPFC	A-F3/C-F4 in A and S	2 mA (sham A)	20 min	0.057 mA/cm^2^	WCST	PE CC	CWG: PE ↓ and CC ↑ after active tDCS intervention and follow-up compared with pre-intervention. CBG: PE ↓ and CC ↑ at post-intervention and follow-up for active tDCS compared with sham group.
Müller et al., 2021 [42]	tDCS	Offline	DLPFC	A-F3/C-F4 in A and S	2 mA (sham B)	20 min	0.08 mA/cm^2^	DCCS	NR	NS
Inhibitory control										
Fecteau et al., 2014 [39]	tDCS	Offline	DLPFC	A-F4/C-F3 in A and S	2 mA (sham C)	30 min	0.057 mA/cm^2^	UGM UGC	AR	UGM: NS UGC: acceptance rate ↓ after active tDCS compared with sham group
Alizadehgoradel et al., 2020 [32]	tDCS	Offline	DLPFC	A-F3/C-F4 in A and S	2 mA (sham A)	20 min	0.057 mA/cm^2^	Go/no-go	ACC RT	CWG: ACC ↑ and RT ↓ for go/no-go trails at intervention and follow-up for active tDCS compared with pre-intervention; CBG: ACC ↑ and RT ↓ for go/no-go trails at intervention and follow-up for active tDCS compared with sham group.
Müller et al., 2021 [42]	tDCS	Offline	DLPFC	A-F3/C-F4 in A and S	2 mA (sham B)	20 min	0.08 mA/cm^2^	Go/no-go	NR	NS
								SST	NR	NS
Vanderhasselt et al., 2020 [26]	tDCS	Offline	DLPFC	A-F4/C-F3 in A and S	2 mA (sham A)	20 min	0.057 mA/cm^2^	Rewarded go/no-go	Go-ACC no-go-ACC	Rewarded condition: go-ACC ↓ after active tDCS compared with sham group; for no-go-ACC, no significant difference between active tDCS and sham group
Verveer et al., 2020a [38]	tDCS	Offline	DLPFC	A-F4/C-F3 in A and S	2 mA (sham A)	T13I20	0.057 mA/cm^2^	Go/no-go	ACC RT	One day after the last interventions: no significance between active tDCS and sham group; Three months after the last interventions: no-go-RT ↓ after active tDCS compared with sham group
								ERP	No-go-N2 Ap; no-go-N3 AP; no-go-ERN AP	One day after the last interventions: no significance between active tDCS and sham group; Three months after the last interventions: no-go-N3 ↓ after active tDCS compared with sham group
Verveer et al., 2020b [31]	tDCS	Online	DLPFC	A-F4/C-F3 in A and S	2 mA (sham A)	T13I20	0.057 mA/cm^2^	Go/no-go	ACC RT	NS
Weidler et al., 2020 [24]	tDCS	Offline	DLPFC	A-F4/C-LSBA in A and S	1.5 mA (sham D)	20 min	A-0.057 mA/cm^2^; C-0.015 mA/cm^2^	SSRT	RT SI-ACC	RT ↓ after active tDCS compared with sham group; successfully inhibited ACC ↑ after active tDCS compared with sham group
Overall executive functioning										
Da Silva et al., 2013 [45]	tDCS	Offline	DLPFC	A-F3/C-RSDA in A and S	2 mA (sham D)	20 min	0.057 mA/cm^2^	FAB	Total score	Total score ↑ after active tDCS compared with sham group.
Klauss et al., 2014 [44]	tDCS	Offline	DLPFC	A-F4/C-F3 in A and S	2 mA (sham D)	13 min	0.057 mA/cm^2^	FAB	Total score	NS
Palacios et al., 2013 [25]	tDCS	Offline	DLPFC	A-F3/C-RSDA in A and S	1 mA (sham D)	10 min	0.029 mA/cm^2^	FAB	Total score	Total score ↑ after active tDCS compared with sham group in individuals classified as Lesch IV (most severe alcohol use) only.

A = anodal; ACC = accuracy; AR = acceptance rate; AP = amplitude; A and S = both active and sham group; C = cathodal; CC = completed categories; CE = center electrode; CWG = comparison within group; CBG = comparison between groups; DLPFC = dorsolateral prefrontal cortex; DCCS = dimensional change card sort; EP = electrodes position; T13I20 = two 13 min interventions with a 20 min break in between; ERP = event-related potential; FAB = frontal assessment battery; ID = reference; OL = online; OFL = offline; RT = reaction time; MACC = memory accuracy; NR = not reported; NS = no significance between active and sham group; PE = perseverative errors; SST = stop-signal task; SSRT = stop-signal reaction-time task; LSBA = left supraorbital area; RSDA = right supradeltoid area; RE = return electrode; SI = successfully inhibited; Sham A = current applied for the first 30 s; Sham B = the current went from 0.3 mA to 2.0 mA and back again at a rate of 0.1 mA per second; Sham C = current applied for the first and last 30 s; Sham D = current applied for the first 20 s; tDCS = transcranial direct current stimulation; T = technique; HD-tDCS = high definition transcranial direct current stimulation; UGC = ultimatum game—cigarettes; UGM = ultimatum game—money; WM = working memory; WCST = Wisconsin card-sorting task; ↑ = increase; ↓ = decrease.

**Table 4:** Summary of findings on attention and alertness.

ID	Protocol							Assessment		Results
	T	Online/Offline	Area	EP	Intensity	Duration	Density	Task and Measures	Indicators	
Sustained Attention and Alertness										
Xu et al., 2013 [40]	tDCS	Offline	DLPFC	A-F3/C-RSBA in A and S	2 mA (sham C)	20 min	0.057 mA/cm^2^	VAT	RT hit rate	NS
Müller et al., 2021 [42]	tDCS	Offline	DLPFC	A-F3/C-F4 in A and S	2 mA (sham B)	20 min	0.08 mA/cm^2^	CPT	NR	NS
Cai et al., 2024 [27]	HD-tDCS	Offline	DLPFC	CE (either A/C)-F3, RE-AF3, F1, F5, and FC3 in A and S	2 mA (sham A)	15 min	NR	WM/Attention task	SACC SRT	Search ACC: no significant difference between active and sham groups. Search RT ↓ after anodal F3 compared with the C-F3 and sham groups.
Attentional Bias										
Meng et al., 2014 [41]	tDCS	Offline	FPT	A-left FPT/C-right FPT; double A-BOL/double C-bilateral FPT; SOPA	1 mA (sham: A)	20 min	NR	Attention task	FC	A declining trend in fixation counts of smoking-related cues was observed following double C-bilateral FPT, tDCS, but the results were not significantly different from sham.
Shahbabaie et al., 2018 [34]	tDCS	Offline	DLPFC	A-F3/C-RS; A-F4/C-LS; A-F3/C-RSBR; A-F4/C-LSR; A-F3/C-F4; SC: one electrode F4/other electrode F3	2 mA (sham A)	T13I20	0.057 mA/cm^2^	PDT	RT DBI EBI	RT ↓, DBI ↓, and EBI ↓ after A-F3/C-right shoulder and A-F3/C-F4 group interventions compared with sham group; no significant difference between other active group and sham group
Motivated Attention										
Khajehpour et al., 2022 [33]	tDCS	Offline	DLPFC	A-F4/C-F3 in A and S	2 mA (sham E)	20 min	0.637 mA/cm^2^	ERP	P3-AP LPP-AP	P3 amplitude ↓ after active tDCS compared with sham group; LPP amplitude: no significant difference between active and sham group;
Palacios et al., 2013 [25]	tDCS	Offline	DLPFC	A-F3/C-RSDA in A and S	1 mA (sham D)	10 min	0.029 mA/cm^2^	ERP	P3-AP	P3 ↑ after active tDCS compared with sham group. P3 ↓ after active tDCS compared with sham group in individuals classified as Lesch II (mild alcohol use).
Da Silva et al., 2013 [45]	tDCS	Offline	DLPFC	A-F3/C-RSDA in A and S	2 mA (sham D)	20 min	0.057 mA/cm^2^	ERP	P3-AP	P3↑ after active tDCS compared with sham group.

Note. A = anodal; ACC = accuracy; AP = amplitude; A and S = both active and sham groups; BOL = bilateral occipital lobe; C = cathodal; CE = center electrode; CPT = continuous performance task; DBI = disengagement bias index; EBI = engagement bias index; ERP = event-related potential; PDT = pictorial probe detection task; DLPFC = dorsolateral prefrontal cortex; EP = electrodes position; T13I20 = two 13 min interventions with a 20 min break in between; FC = fixation count; FPT = frontal–parietal–temporal association area; ID = reference; LS = left shoulder; LSR = left supraorbital ridge; NR = not reported; active and sham group; RE = return electrode; RS = right shoulder; RSBR = right supraorbital ridge; RSBA = right supraorbital area; RSDA = right supradeltoid area; RT = reaction time; SC = sham condition; Sham A = current applied for the first 30 s; Sham B = the current went from 0.3 mA to 2.0 mA and back again at a rate of 0.1 mA per second; Sham C = current applied for the first and last 30 s; Sham D = current applied for the first 20 s; Sham E = the simulator was turned off after a gradual ramping up of electrical current to 2 mA and down to 0 mA for the first and last 60 s; T = technique; SOPA = sham one of the placements above; VAT = visual attention task; ↑ = increase; ↓ = decrease.

**Table 5:** Summary of findings on risk taking.

ID	Protocol							Assessment		Results
	T	Online/Offline	Area	EP	Intensity	Duration	Density	Task and Measures	Indicators	
Boggio et al., 2010 [28]	tDCS	Online	DLPFC	A-F4/C-F3; A-F3/C-F4; SOPA	2 mA (sham A)	15 min	0.057 mA/cm^2^	Risk task	CLRHRP-TPE-RT	F3/C-F4: risk taking ↑ and A-F4/C-F3: risk taking ↑ compared with sham group.
Pripfl et al., 2013 [29]	tDCS	Online	DLPFC	A-F1, F3, AF1/C-F4; A-F2, F4, AF2/C-F3; SOPA	0.45 mA (sham C)	15 min	A-0.085 mA/cm^2^; C-0.013 mA/cm^2^	HCCT	NOCC	Number of cards chosen ↓ after A-F2, F4, AF2 interventions compared with A-F1, F3, AF1 and sham group.
								CCCT	NOCC	Number of cards chosen ↓ after A-F1, F3, AF1 interventions compared with A-F2, F4, AF2 and sham group.
Fecteau et al., 2014 [39]	tDCS	Offline	DLPFC	A-F4/C-F3 in A and S	2 mA (sham A)	30 min	0.057 mA/cm^2^	RTM RTC	Choice of low-risk vs. high-risk options	Risk task—money: no significance between active and sham group; risk task—cigarettes: no significance between active and sham group.
Gorini et al., 2014 [36]	tDCS	Offline	DLPFC	A-F3/C-F4; A-F4/C-F3; SOPA	1.5 mA (sham A)	20 min	0.047 mA/cm^2^	BART	Average number of pumps on an unexploded ballon	Average number of pumps on an unexploded ballon ↓ after A-F3 or A-F4 group compared with the baseline test; no significant difference from the sham group compared with baseline test.
								GDT	Average number of conservative bets	Average number of conservative bets ↑ after A-F3 intervention compared with sham group; average number of conservative bets ↓ after A-F4 intervention compared with baseline test.
Alizadehgoradel et al., 2020 [32]	tDCS	Offline	DLPFC	A-F3/C-F4 in A and S	2 mA (sham A)	20 min	0.057 mA/cm^2^	BART	AV MNP	CWG: AV ↓ and MNP ↓ after active tDCS intervention and follow-up compared with pre-intervention; CBG: AV ↓ and MNP ↓ at post-intervention and follow-up for active tDCS compared with sham group.
Verveer et al., 2020b [31]	tDCS	Online	DLPFC	A-F4/C-F3 in A and S	2 mA (sham A)	T13I20	0.057 mA/cm^2^	TCGT	Proportion of high-risk choices; average points earned	NS

Note. A = anodal; AV = adjusted value; AAT = approach-avoidance task; A and S = both active and sham groups; BART = balloon analogue risk task; BIS-11 = Barratt impulsiveness scale version 11; C = cathodal; CCCT = cold Columbia card task; CLRHRP-TPE-RT = choice between low-risk and high-risk prospect and total points earned during the risk task; DLPFC = dorsolateral prefrontal cortex; DDT = delay-discounting task; EP = electrodes position; FAB = frontal assessment battery; GDT = game-of-dice task; KDDT = Kirby delay-discounting task; HCCT = hot Columbia card task; IAT = implicit association test; mTAP = modified Taylor aggression paradigm; ID = reference; MNP = maximum number of pumps; NOCC = number of cards chosen; NS = no significant difference between active and sham group; OL = online; OFL = offline; RT = reaction time; RTM = risk task—money; RTC = risk task—cigarettes; RSBA = right supraorbital area; Sham A = current applied for the first 30 s; Sham B = the current went from 0.3 mA to 2.0 mA and back again at a rate of 0.1 mA per second; Sham C = current applied for the first and last 30 s; Sham D = current applied for the first 20 s; Sham E = the simulator was turned off after a gradual ramping up of electrical current to 2 mA and down to 0 mA for the first and last 60 s; SOPA = sham one of the placements above; T = technique; TCGT: two-choice gambling task; T13I20 = two 13 min interventions with a 20 min break in between; ↑ = increase; ↓ = decrease.

**Table 6:** Summary of findings on other cognitive functions.

ID	Protocol							Assessment		Results
	T	Online/Offline	Area	EP	Intensity	Duration	Density	Task and Measures	Indicators	
Impulsivity and Self-Control										
Eskandari et al., 2021 [37]	tDCS	Offline	DLPFC	A-F3/C-F4; A-F4/C-F3; SOPA	2 mA (sham: NR)	20 min	NR	BIS-11	Total score	Total score ↓ after A-F3/F4 compared to sham group
Müller et al., 2021 [42]	tDCS	Offline	DLPFC	A-F3/C-F4 in A and S	2 mA (sham B)	20 min	0.08 mA/cm^2^	DDT	NR	NS
Gaudreault et al., 2022 [35]	tDCS	Offline	DLPFC	A-F4/C-F3 in A and S	2 mA (sham C)	20 min	0.057 mA/cm^2^	KDDT	K value	CBG: no significance between active and sham group; CWG: K value ↓ after active and sham tDCS interventions compared to baseline test
Motivation and Willingness										
den Uyl et al., 2015 [43]	tDCS	Offline	DLPFC IFG	A-F3/C-RSBA; A-right IFG/C-LSBA; SOPA	1 mA (sham A)	10 min	0.029 mA/cm^2^	Affective IAT	RT	RT ↓ after A-F3 compared with A-right IFG and sham group.
Gaudreault et al., 2022 [35]	tDCS	Offline	DLPFC	A-F4/C-F3 in A and S	2 mA (sham C)	20 min	0.057 mA/cm^2^	CL	Total score	After the last session, the total score post-test was significantly higher in the active tDCS group than at baseline; one month after last stimulation: no significant difference from baseline.
den Uyl et al., 2017 [30]	tDCS	Online	DLPFC	A-F3/C-F4 in A and S	2 mA (sham A)	20 min	A-0.057 mA/cm^2^; C-0.02 mA/cm^2^	AAT	AB	NS
Reactive Aggression										
Weidler et al., 2020 [24]	tDCS	Offline	DLPFC	A-F4/C-LSBA in A and S	1.5 mA (sham D)	20 min	A-0.057 mA/cm^2^; C-0.015 mA/cm^2^	mTAP	OSPS	NS
Basic Response Time										
Müller et al., 2021 [42]	tDCS	Offline	DLPFC	A-F3/C-F4 in A and S	2 mA (sham B)	20 min	0.08 mA/cm^2^	SRTT	RT	NS

Note. A = anodal; AB = approach bias; A and S = both active and sham groups; BIS-11 = Barratt impulsiveness scale version 11; C = cathodal; CL = contemplation ladder; DLPFC = dorsolateral prefrontal cortex; DDT = delay-discounting task; EP = electrodes position; KDDT = Kirby delay-discounting task; ID = reference; IAT = implicit association test; IFG = inferior frontal gyrus; AAT = approach-avoidance task; LSBA = left supraorbital area; mTAP = modified Taylor aggression paradigm; NR = not reported; NS = no significant difference between active and sham groups; OL = online; OFL = offline; OSPS = opponent’s punishment selection; RT = reaction time; RSBA = right supraorbital area; SOPA = sham one of the placements above; SRTT = simple reaction-time task; Sham A = current applied for the first 30 s; Sham B = the current went from 0.3 mA to 2.0 mA and back again at a rate of 0.1 mA per second; Sham C = current applied for the first and last 30 s; Sham D = current applied for the first 20 s; T = technique; ↑ = increase; ↓ = decrease.

## Data Availability

Data sharing is not applicable to this article as no new data were created or analyzed in this study.
